# Unearthing the role of septins in viral infections

**DOI:** 10.1042/BSR20231827

**Published:** 2024-03-06

**Authors:** Jasmine Elanie Khairat, Muhammad Nur Adam Hatta, Nurshariza Abdullah, Adzzie Shazleen Azman, Shee Yin Ming Calvin, Sharifah Syed Hassan

**Affiliations:** 1 Institute of Biological Sciences (ISB), Faculty of Science, Universiti Malaya, Kuala Lumpur 50603, Malaysia; 2 Jeffrey Cheah School of Medicine and Health Sciences, Monash University Malaysia, Bandar Sunway 47500, Selangor, Malaysia; 3 School of Health Sciences, Universiti Sains Malaysia, Kubang Kerian 16150, Kelantan, Malaysia; 4 School of Science, Monash University Malaysia, Bandar Sunway 47500, Selangor, Malaysia

**Keywords:** diseases, host-viral interaction, roles, septin

## Abstract

Septin proteins are a subfamily of closely related GTP-binding proteins conserved in all species except for higher plants and perform essential biological processes. Septins self-assemble into heptameric or octameric complexes and form higher-order structures such as filaments, rings, or gauzes by end-to-end binding. Their close association with cell membrane components makes them central in regulating critical cellular processes. Due to their organisation and properties, septins function as diffusion barriers and are integral in providing scaffolding to support the membrane’s curvature and stability of its components. Septins are also involved in vesicle transport and exocytosis through the plasma membrane by co-localising with exocyst protein complexes. Recently, there have been emerging reports of several human and animal diseases linked to septins and abnormalities in their functions. Most of our understanding of the significance of septins during microbial diseases mainly pertains to their roles in bacterial infections but not viruses. This present review focuses on the known roles of septins in host–viral interactions as detailed by various studies.

## Background

Host–pathogen interactions are imperative to our understanding of infectious diseases, their treatment and prevention. The mechanisms by which pathogens invade and proliferate in the hosts can be explained by investigating and examining the various stages of infection. The most recent review on septins discussed in length the history of its biology since its discovery and its significance in bacterial infections [1], while another review deliberated about its general role in microbial infection with emphasis only on present insights on the rice blast fungus, the vaccinia virus, and *Shigella flexneri* [2]. Septins have been shown to have important roles in host cell defence against bacterial infection. One of the ways septins can impact bacterial infections is by forming a physical barrier that can limit bacterial spread. For example, in the case of *S. flexneri*, a bacterium that causes bacillary dysentery, Mostowy et al. showed that septins can form a cage-like structure around bacterial pathogens, immobilizing and preventing them from spreading to neighbouring host cells by interfering with the actin-polymer tails development [3]. The interaction of bacteria caging by septins in bacterial infection is shown to increase the efficiency of autophagy reaction [4,5].

The role of septins during bacterial infection has been extensively reviewed before, but to the best of our knowledge, only one review was published in 2021 on the interplay between septins and viral infections. Nonetheless, that particular paper did not include the more recent studies that are discussed here. With the emergence of new and resistant viruses, it is urgent to understand how pathogens interact with their hosts for successful invasion. In this review, we present several examples of the potential involvement of septins in viral infections to highlight what is known and unknown about the interplay between septin proteins and viruses. It is intended for the interest of members of several fields of research, such as virology, molecular biology and structural biology, to aid in the discovery of appropriate preventive strategies and spur the development of therapeutic measures against virus diseases.

## Septins

**Figure 1:**
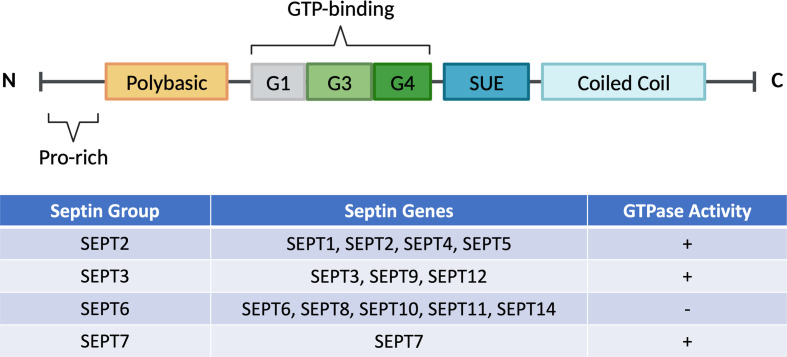
Schematic diagrams of typical septin sequence with its classification and GTPase activity Highly conserved GTP-binding domains consist of G1, G3, and G4 motifs, polybasic region, and septin unique elements (SUE) are present in mammalian septins. All septins have various lengths and amino acid sequences of the N-terminal (amino-terminal) and C-terminal (carboxy-terminal). Commonly, septins are classified into four distinct groups: SEPT2 (SEPT1, SEPT2, SEPT4, SEPT5), SEPT3 (SEPT3, SEPT9, SEPT12), SEPT6 (SEPT6, SEPT8, SEPT10, SEPT11, SEPT14), and SEPT7. Almost all septins possessed a slow intrinsic GTPase activity except for the SEPT6 group. This is due to the absence of Thr78 residue that prevents hydrolysis of GTP to GDP in the SEPT6 group.

**Figure 2:**
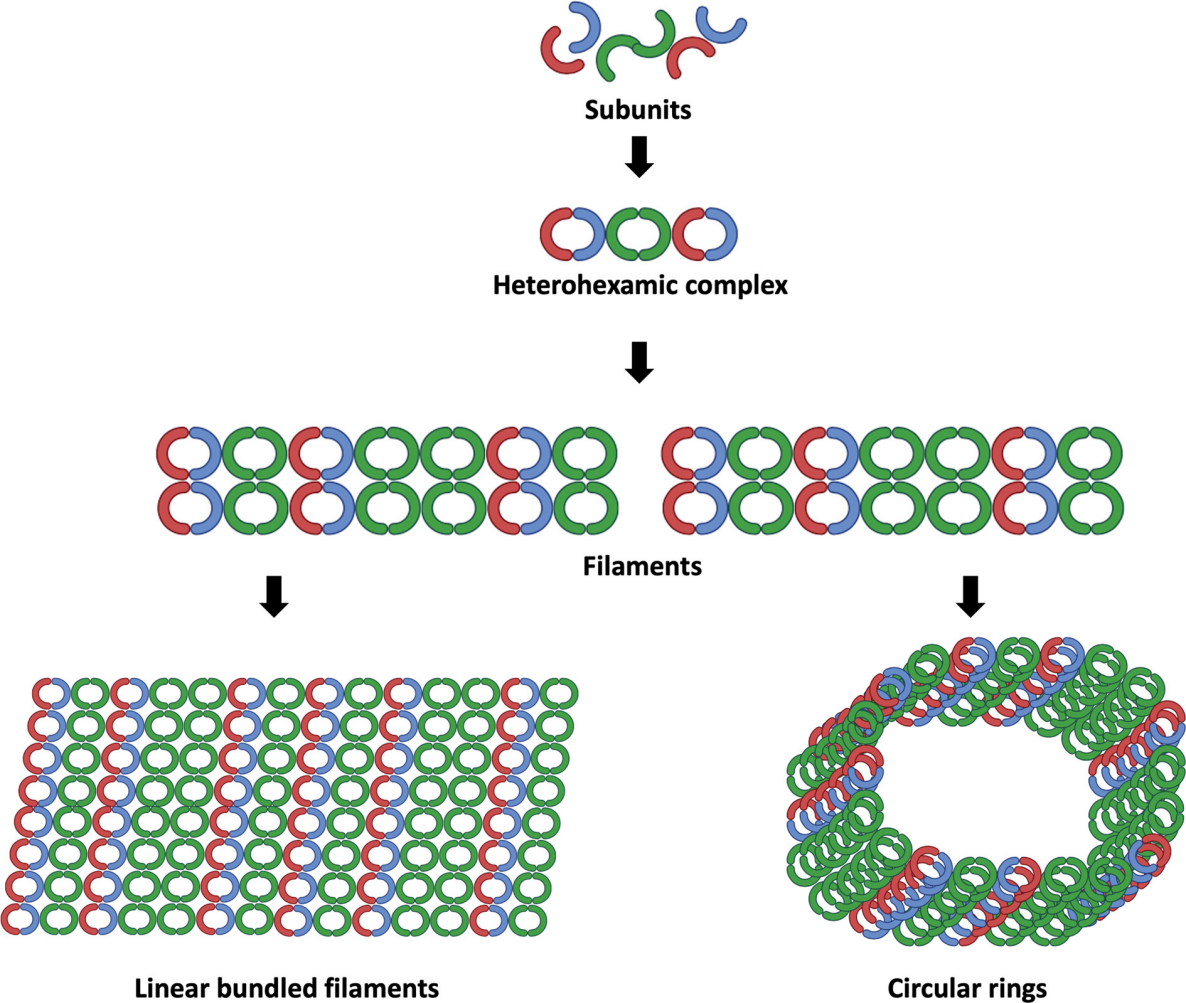
Cytoskeleton dynamics of septins GTP-binding domain interface and the carboxy-terminal NC interface of septin subunits interact creating complexes that link end-to-end to form filaments. Septin from several groups are shown in various colours. In humans, it has been demonstrated that septins form complexes with two, three, and/or four septin subunits (canonical and non-canonical complexes). Septin oligomers will undergo polymerization to form a high-order filamentous structure. Later, septin hetero-oligomers establish non-polar filaments that can link up and generate linear bundles filaments and circular ring-like structures.

Initial studies of septins started around 50 years ago when they were first discovered during a screening of temperature-sensitive mutations in the budding yeast, *Saccharomyces cerevisiae* [6]. It was revealed that these relatively unknown proteins are crucial in the separation of the budding yeast [7,8]. As a family of cytoskeletal proteins on the plasma membrane, it forms complexes and polymerises into filaments that can bind to the membrane along with other cytoskeletal components such as actin and microtubules [9,10]. These proteins are broadly conserved across species and are involved in a multitude of processes, including regulation of cytokinesis, exocytosis, control of cell cycle, diffusion barrier for proteins, vesicle trafficking, and maintenance of cell polarity, to name a few [11–13]. To date, all 13 human septins are classified into four separate groups; SEPT2, SEPT3, SEPT6 and SEPT7 [14,15], based on their sequence homology (Figure 1). Classifying mammalian septins into four subgroups is essential because individual septins from each group can form septin complexes and polymerise into various higher-order structures, such as filaments, bundles or rings (Figure 2) [8,9]. The interaction of septins with membrane lipids and cytoskeletal components such as microfilaments of actin or microtubules may further affect their filament polymerisation. This indicates that septins assembly depends not only on its biochemical properties and post-translational changes but also on its association with binding partners, intracellular structures, and organelles. The expression of some septin isoforms is ubiquitous in the host (SEPT2, SEPT7 and SEPT9), some are extensively expressed in most tissues (SEPT4, SEPT8, SEPT10 and SEPT11), while other isoforms exhibit tissue-specific expression [16,17].

Septin complexes act as plasma membrane scaffolds that promote functional protein–protein interactions of membrane-bound proteins and also function to compartmentalise distinct cell domains [18,19]. Septins scaffolding is also crucial in the regulation of vesicle fusion [20], microtubule-dependent transport in the cytoplasm [7], during host–pathogen interactions, and autophagy [3,21,22]. As more and more about their tissue-specific functions are still being studied, their contribution to various organs’ growth, maintenance, and diseases has been widely examined [8]. This includes the role of septin isoforms in different types of cancer (leukaemia, lymphoma, melanoma, breast, colon, and ovarian) [23–29] and neurological disorders (Alzheimer’s disease, Parkinson’s disease, Down’s syndrome, and schizophrenia) [19,30,31] has already been proven. Recently, new insights have emerged on the role of septins in viral infections other than their more documented roles in cell integrity, cytokinesis, and bacterial infections.

## Septins in viral infections

**Figure 3:**
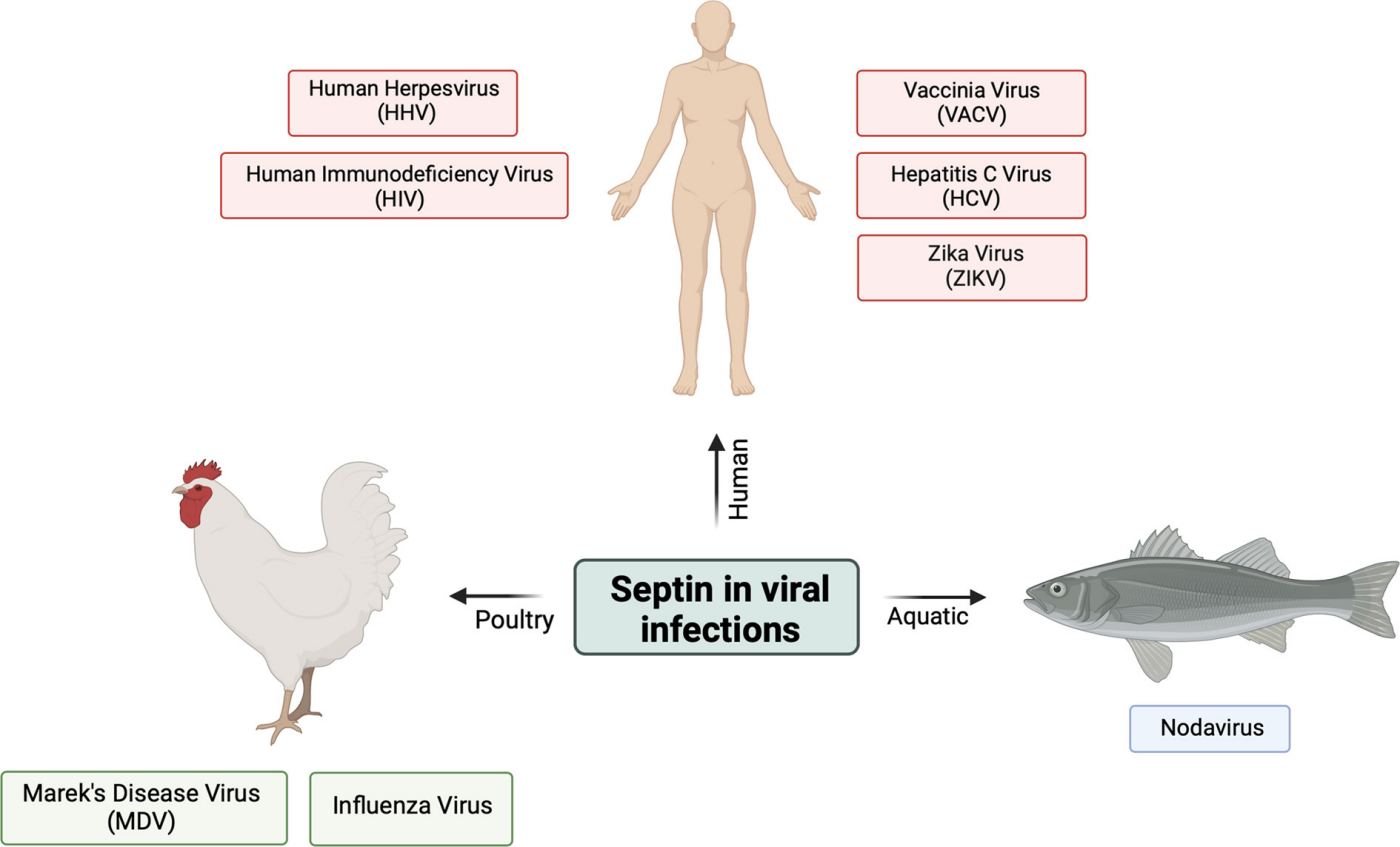
Septin interactions in viral pathogenesis described in this review This review focuses on eight pathogenic viruses that are associated with septin functions during pathogenesis in humans (Vaccinia Virus, VACV; Hepatitis C Virus, HCV; Human Herpesvirus, HHV; Zika Virus, ZIKV; Human Immunodeficiency Virus, HIV), aquatic animals (Nodavirus), and poultry animals (Marek's Disease Virus, MDV; Influenza Virus).

Septins have been associated with the pathogenesis of various microbial agents such as bacteria, fungi, and viruses. However, most of these studies have been centred on bacterial infections, whereas our understanding of its role in viral infections is still limited. Nevertheless, new research has emerged to shed light on septins’ part in viral infections. Viruses are microscopic infectious agents that are obligate intracellular microorganisms and follow several life cycle stages to infect their hosts. Understanding the role of septins in various stages of viral replication may improve our knowledge of host-pathogen interactions, which allows for the creation of new therapeutics against viral infections. This review will describe eight infectious viruses and their septin(s) interactions upon viral pathogenesis in human, aquatic animals, and poultry animals (Figure 3).

### Vaccinia virus

Much of our understanding of virus-cell membrane interactions has been based on extensive studies on vaccinia virus (VACV), a double-stranded DNA virus that belongs to the *Orthopoxviridae* family [32]. Poxviruses are DNA viruses that go through their entire replication, including genome transcription and assembly in the host cytoplasm. Screening of human genome-wide RNAi showed that SEPT1 and SEPT9 had been reported to play a role in limiting the spread of poxvirus in infected cells and that knockdown of these genes increased viral replication [33]. Extraction of infected HeLa cells showed that SEPT9 co-purifies with SEPT2, SEPT7, and SEPT11, which suggests the proteins formed a functional complex [34] and depletion of any member of these septins led to a significant increase in viral load [33]. In another screen, Sivan et al. [35] identified host factors interacting with virion proteins during VACV replication. After knocking down 530 genes, SEPT11 was one of the genes associated with an increase in virus spread; however, the capacity at which the protein is involved in the pathogenesis is largely unknown. During the VACV release stage, some viruses will remain attached to the outside of the cell as cell-associated enveloped viruses (CEV) [33,35]. This allows the virions to signal to host cells to induce actin polymerisation to assist in cell-to-cell spread [36,37]. It was noted that during this event, the CEVs were also surrounded by septin proteins, namely SEPT2, SEPT6, SEPT7, and SEPT9, which were recruited to the plasma membrane to inhibit its release [38]. The same study also showed that upon RNA interference-mediated septin depletion, there was an increase in actin tails’ formation and virus release. This study highlighted the significant role of septin proteins and other membrane proteins in inhibiting viral release.

### Nodavirus

Since there is a lack of attention to the effect of viruses on aquatic organisms, little is understood about fish's mechanism to combat viral infections. In fish aquaculture, outbreaks of viral infections are usually caused by Nodavirus [39,40]. Fish nodaviruses are non-enveloped small RNA viruses that belong to the *Nodaviridae* family under the β-nodavirus group. They are also commonly known as Nervous Necrosis Virus (NNV), as infected fishes often exhibit neurological disorders that lead to abnormal swimming behaviour and lethargy [41,42]. Ghiasi et al. [43] presented that the virus can be detected in many organs, primarily the central nervous system, including the brain, spinal cord, and retina. The impact of the virus on the fish industry has caused a massive economic loss worldwide, with a high mortality rate of 100% in juvenile fishes and larvae. In the brain, multiple genes have been detected to be differentially expressed upon infection by using the suppression subtractive hybridisation method [44]. This study was the first to observe an up-regulation of SEPT8 only in the non-infected control brain but not in the nodavirus-infected brain of sea bream. This septin protein belongs to the immune-related cluster genes, heat shock proteins (Hsp-70), Fms interacting protein, TNF-α induced protein, and interferon protein. The results appear to indicate the involvement of SEPT8 in regulating the immune response of the fish during infection, but the difference in expression was not studied further. Nervetheless, it could have a potential role in the viral pathogenesis in the brain as SEPT8 are abundant in this organ especially in mammals [16,45,46]. However, the biological role of SEPT8 in fish remains unknown as experimental proof is lacking; therefore, conclusive interpretations from the study could not be made.

### Marek’s disease virus

For poultry animals, Marek’s disease (MD) is one of the leading causes of viral infections reported in flocks [47]. The causative agent is Marek’s disease virus (MDV), alternatively named Gallid alphaherpesvirus 2 (GaHV-2), which is characterised by lymphoid tumours, neurological disorders, and immunosuppression. Currently, the control of this viral disease remains a challenge due to the evolution of virulence which leads to the emergence of hypervirulent pathotypes of this cell-associated oncogenic virus [48]. Therefore, understanding the host–virus interactions is crucial, particularly during the latency and virus reactivation periods, which eventually will transform infected CD4+ T cells into lymphomas in infected birds. The MD vaccines effectively prevent infection, halt the development of tumours, and reduce immunosuppression and paralysis, which have saved the poultry industry from this devastating disease [49]. However, host interactions with the virus have been poorly understood due to limited studies available. In one of the studies of infected chicken's thymuses, 119 differentially expressed proteins were identified in post-infection with Marek’s virus, with 20 proteins directly linked to viral infection and replication, immunity, apoptosis, and tumour growth [50]. Two septin proteins, SEPT6 and SEPT9, were identified for the first time through mass spectrometry. These proteins were found to be significantly decreased upon infection and have been implicated in oncogenesis by promoting tumour progression through the activation of the hypoxia-inducible factor-1 pathway [25]. More in-depth studies on this family of septins should be done to recognise its role in viral oncogenesis.

### Influenza virus

Another debilitating disease in poultry is the highly pathogenic avian influenza virus (HPAI) infection which has caused billions of dollars in monetary losses in affected countries. Infected chickens developed severe central nervous system dysfunction and classic nonsuppurative encephalitis. During H5N1 infection, 23 host’s genes are controlled by known genes which are important factors affecting the cytoskeletal process, the transduction and proliferation of the neural signal and the folding of protein during stress [51]. One up-regulated gene isolated from HPAI H5NI-infected brain tissues was the SEPT5 gene [51,52]. The up-regulation of SEPT5 prompted the accumulation of SEPT5 protein which has been reported to be involved in the pathogenesis of Parkinson’s disease caused by the loss of parkin protein activity in the dopaminergic neurons [53]. In another study, a pull-down assay was performed to study the host’s interacting or binding proteins in the brains of H5N1-infected chickens with recombinant SEPT5 protein to understand the role of septin proteins in avian influenza pathogenesis [54]. Using mass spectrometry, other septins were identified to be directly or indirectly involved in binding to viral proteins such as SEPT2, SEPT6, SEPT7, and SEPT11, along with collapsing response mediator protein 2 (CRMP2), tubulin proteins, and heat-shock proteins (HSP). However, the specific mechanism of binding or interaction between these complexes of septins was not further studied. These studies indicated the possibility that septin proteins have significant roles in the neuropathological progress of the chicken brain tissues during HPAI virus infection.

### Hepatitis C virus

In the case of Hepatitis C, the etiologic agent, Hepatitis C virus (HCV), is a blood-borne virus and causes diseases ranging from acute to chronic hepatitis. If left untreated, chronic patients may develop cirrhosis or liver cancer, a severe health complication that burden the healthcare system [55–57]. Several cellular proteins have been studied that facilitated HCV replication, but the molecular basis of HCV replication and proliferation in hosts remains to be investigated. During HCV infection, it has been reported that SEPT6 interacts with NS5b viral proteins by forming a complex with the host's hnRNP A1 proteins through co-immunoprecipitation and yeast two-hybrid studies [58]. Additionally, HCV replication was inhibited with SEPT6 knockdown and overexpression of an N-terminal truncated SEPT6. Encoded by the HNRNPA1 gene, the HnRNP A1 protein is the host’s RNA-binding protein, which is involved in multiple RNA processes [59] such as packaging pre-mRNA [60], splicing [61,62] and transport of molecules from the nucleus to the cytoplasm [63]. This demonstrates that the binding of the host’s hnRNP A1 to SEPT6 played crucial roles in HCV replication by forming a complex with viral NS5b protein and viral RNA. SEPT6 can also act as a scaffolding molecule involved in the localisation of the replication complex to a membranous compartment in the cell and recruited host proteins that promote replication of HCV RNA to the replication complex. In the same study by Kim et al. [58], there was also an increase in co-immunoprecipitation of SEPT2 with SEPT6 during infection. This may be due to a rise in septins polymerisation and interaction with viral NS5b in HCV-infected cells, facilitating viral replication. In HCV infection, the risk of developing liver cirrhosis is measured by the accumulation of lipid droplets (LDs). From a transcriptomic analysis study, most septins expressions were up-regulated in HCV-induced cirrhosis compared to normal liver, except SEPT10. Notably, a significant upregulation of SEPT9 and assembly into filaments was observed [64]. As septin filaments contain different septin isoforms, this is in line with the staining using SEPT2 and SEPT9 antibodies that indicated co-localisation of the two proteins. The effects of SEPT9 on LDs depend on the binding of phosphatidylinositol, which then regulates the development of septin filamentous structures and their association with microtubules. The same interactions may be present in other hepatitis viruses that cause liver cirrhosis, which is worth looking at in the future.

### Human herpesvirus

Many evidence indicates that knowledge of septins is essential for understanding the pathogenesis and management of infectious diseases, as they impact both host and pathogen proteins. In the case of Human herpesvirus 8 (HHV-8), the virus has been linked to Kaposi’s sarcoma and other human malignancies, such as AIDS-associated multicentric Castleman's disease [65]. Nevertheless, the mechanism of the molecular pathology of HHV-8 is not yet fully understood due to limited studies on host–pathogen protein interactions, which could aid in the treatment and prevention of these diseases. One HHV-8 viral gene with oncogenic potential, Kaposin A [66], has been identified and shown to cause tumorigenic transformation [67], subsequently inducing transformative behaviour of nuclear receptor coactivators in host cells to favour HHV-8 malignancies [68]. To this day, only one study has successfully identified the septin binding partner of Kaposin A. In a phage display library experiment, a Kaposin A-interacting SEPT4 variant was identified and was discovered to induce cell rounding, activating caspase-3, and up-regulate transcriptional factor NF-κB. Co-immunoprecipitation and confocal imaging studies further verified the binding specificity and co-localisation of the Kaposin A protein to the host’s SEPT4 variant. This interaction may play a role in the pathogenesis of human diseases associated with HHV-8, which is the hallmark of viral infections. Other than Kaposin A, the HHV8 genome also codes for proteins that can elicit cellular signalling pathways, potentially leading to the expression of inflammatory and angiogenic molecules known to play an essential role in viral replication and pathogenesis [69]. One of these proteins is the non-structural membrane protein, pK15, predominantly expressed during the HHV8 lytic life cycle *in vitro*. This protein has been shown to recruit multiple cellular proteins, including phospholipase Cγ1, phosphatidylinositol 3-kinase PI3K-C2α, components of the NF-κB pathway, and the Src family of non-receptor tyrosine kinases, and activates different biological processes, including angiogenic and inflammatory pathways [70]. A co-immunoprecipitation study and label-free quantitative mass spectrometry identified novel cellular binding proteins that interact with pK15 from HHV8-infected endothelial cells [71]. It was discovered that SEPT2 and SEPT9 were among the 75 potential pK15-interacting proteins upon induction of the HHV8 lytic cycle, which signalled the involvement of actin cytoskeleton reorganisation. These interactions are in tandem with the role of pK15-mediated endothelial spindle cell formation, the induction of angiogenesis and the promotion of invasiveness in KSHV-infected endothelial cells, contributing to the pathogenesis of the virus.

### Zika virus

With the re-emergence of the Zika virus (ZIKV) infection as an epidemic disease, multiple global efforts have been made to study and understand the associated pathogenesis of this viral infection. ZIKV infection has been notably linked to congenital foetal disabilities during pregnancy [72–74], but the mechanisms by which ZIKV causes microcephaly are poorly understood. Studies on the effects of ZIKV on neuronal cells have been performed with multiple cellular proteins associated with ZIKV structural and non-structural proteins that facilitate viral pathogenesis [75–77]. Since the virus targets neural stem cells in the brain, the viral proteins could impact the function of the brain proteins involved in neuronal cell division. The non-structural proteins are fascinating to researchers as they are responsible for viral replication, assembly, and immune evasion [78,79]. A recent study by Li et al. [80] found that ZIKV protease, specifically NS2B3, was involved in causing delayed cytokinesis in the neural stem cells. The viral protease interacts and cleaved host SEPT2, disrupting the SEPT2/SEPT7 complex at the midbody during cytokinesis. This disruption and reduced expression of both septins alter the septin cytoskeletal components. To reverse this effect, a non-cleavable SEPT2 was introduced into cells expressing the NS2B3, which partially restores the cytokinesis process. The results of this study are the first of their kind and suggest a potential development for antiviral drugs targeting the viral protease to combat the disease. As ZIKV belongs to the flavivirus family, its protease and that of the dengue virus is closely related; however, no studies have discovered if proteases from the same family target septin proteins. Since ZIKV is the only flavivirus known to cause neurological deficits through its ability to penetrate the placenta and foetal brain [80–82], it is highly likely that the affinity towards SEPT2 is unique to ZIKV, which may contribute to its microcephaly mechanisms. Consequently, further investigation into the role of SEPT2 in ZIKV pathogenesis is warranted.

### Human immunodeficiency virus

Since 1981, AIDS has been categorized as a pandemic and endemic disease that has taken approximately 35 million lives worldwide. From the UNAIDS report for 2022, an estimated 4,000 people were infected by the human immunodeficiency virus (HIV) daily [83]. Combating HIV infections has been a challenge to researchers due to the virus’s sophisticated infection mechanisms, which manage to evade the host’s immune system. Targeting the CD4+ T cells weakens the host's immune response towards any infections, leading to immunocompromise or AIDS state when the T cell level falls below 200 copies. Transcriptomic data by Morou et al. shows a 26-fold increase of SEPT9 expression in CD4+ T cells upon this retroviral infection, which is later associated with the regulation of cell cycle and cell proliferation [84]. A study on a South African cohort of AIDS patients with tuberculosis elucidated 40 differentially expressed genes via RNA microarray data set analysis [85]. Gene ontology analysis shows that these genes are associated with organelle formation, signal transduction, and the immune system. Among them, one septin protein subtype, SEPT7, plays crucial roles in cytokinesis, mitosis, spine morphogenesis, neuronal dendrite growth, and structural constituents in sperm production for humans and mice [86]. This is the first study reported on the association of SEPT7 with HIV-related infections. HIV infections have been classified as sexually transmitted infections that can be transferred to a new host via the exchange of body fluids, including semen. A study conducted by Wu et al. revealed that HIV might establish the testis as a primary reservoir since the early stages of infections, even when antiretroviral treatment was administered [87]. Disturbance of cytoskeleton organization occurred at an early stage, leading to an increase in the permeability of the blood–testis barrier. The coculture of HIV's virulence protein, Tat protein, was found to perturb the organization of SEPT7 within the septin-based cytoskeletal components. Additionally, the vimentin-based intermediate filament was also disrupted, causing it to lose its ability to stretch across the Sertoli cell cytosol [87]. Nonetheless, it is important to note that the septin family is affected by HIV infections, and further study should be conducted to confirm its role in the pathogenesis.

## Conclusion

At present, we are only at the tip of the iceberg in understanding the function of septins in the pathogenesis of microbial agents, including viruses, bacteria, and fungi. We have only just begun to discover the role of septins in normal and diseased states, with a more detailed and in-depth study required to unearth the complex pathways involved. We are also in dire need of accurate models to present new insight and clues on septins’ role in infections.

**Figure 4:**
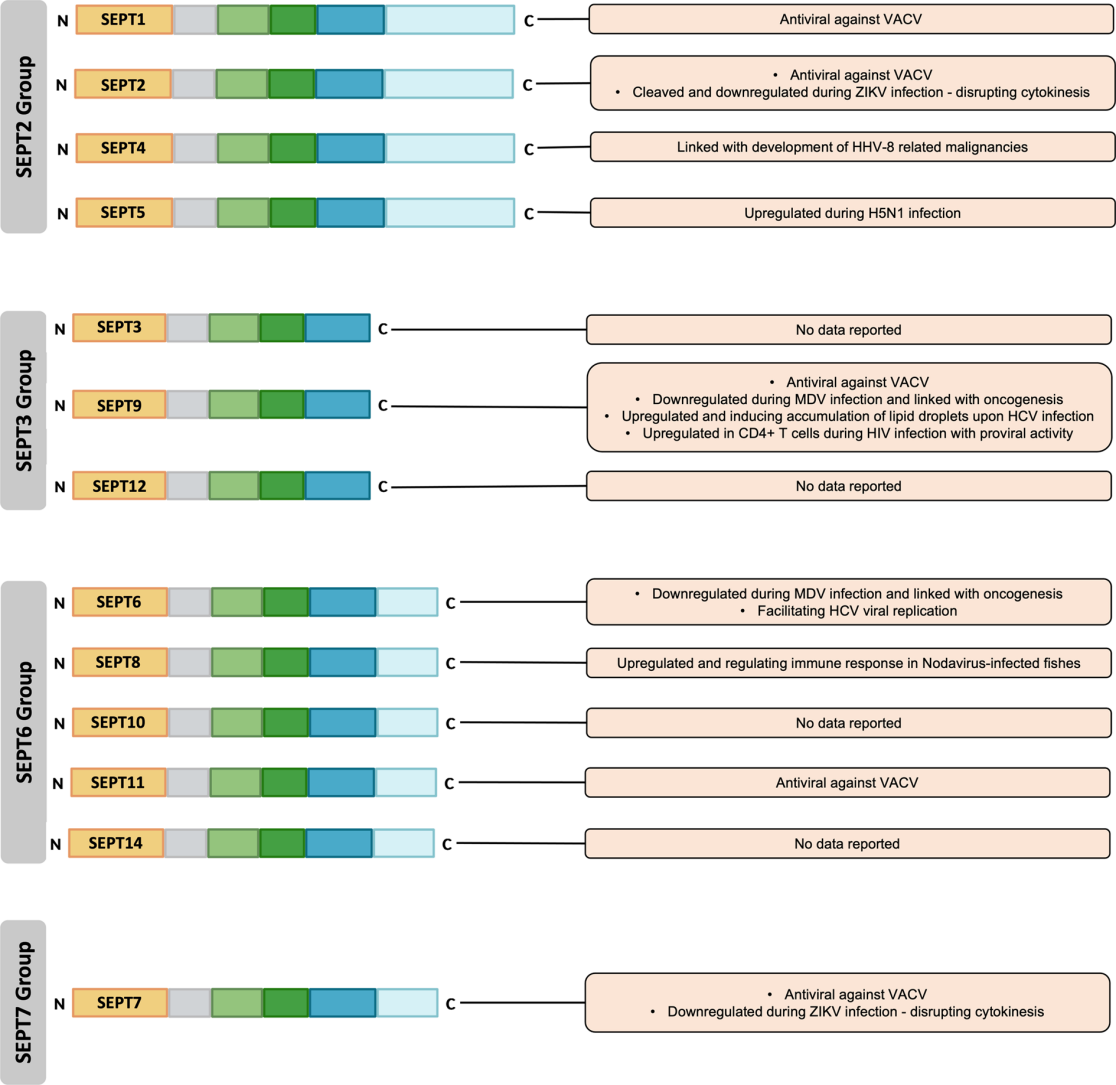
Overview of the role of septins in viral infections SEPT1, SEPT2, SEPT4, SEPT5, SEPT6, SEPT7, SEPT8, SEPT9, and SEPT11 possess antiviral and/or proviral potential depending on the type of viral infections. However, there is a lack of information on the viral interaction of SEPT3, SEPT10, SEPT12, and SEPT14.

The diversity of septins allows for multiple involvements with various cellular biology processes, and changes in their expression levels may alter these processes. In this review, we have discussed how a specific septin subtype may have an antiviral role by limiting viral replication [35] and inhibiting viral load release from the infected cells [35–37], as elucidated in the context of the vaccinia virus. There is also evidence suggesting that septins can have a proviral effect on the viral replication process as it interacts with the host’s proteins to form a functional complex [60]. Nevertheless, the exact role of some septins in viral replication is still undetermined [33,60]. Since septin complexes are highly dynamic and can form complex higher-order fibrillar structures, it is imperative to document the expression patterns of each septin, along with their transcripts, isoforms and gene expression regulation. Overall, the role of septin can be both antiviral and proviral, depending on the specific viral species and/or target cells (Figure 4). To gain a better understanding of how septins influence viruses, it is necessary to confirm their involvement in the replication cycle and discover the mechanisms by which septins and their spliced variants may impact the virus.

Septins may have a role in various stages of the viral replication cycle. Septin scaffolding platforms could be crucial for receptor recruitment to the cell surface during viral docking. Pathogen invasion activates a variety of cellular defensive systems, and one approach involved the aggregation of septin filaments around infectious particles [57]. By scaffolding the autophagy machinery around the pathogen, these structures may aid in the removal of the infectious agent [35]. The list in Table 1 highlighted the known binding proteins of septins associated with viral infectious diseases.

**Table 1:** Septin binding proteins and their role in viral infectious diseases

Septin protein(s)	Type of virus	Binding proteins		Putative role in infectious disease	References
		Host	Viral		
SEPT1	VACV	n/d	n/d	Antiviral against VACV	[33]
SEPT2	VACV	SEPT9, SEPT7 & SEPT11 (Complex)	n/d	Antiviral against VACV	[33]
	ZIKV	SEPT7	NS2B3	Cleaved and down-regulated during ZIKV infection – disrupting cytokinesis	[80]
SEPT4	HHV-8	n/d	Kaposin A pK15	Interaction of Kaposin A with SEPT4 is associated with the development of HHV-8-associated malignancies	[69]
SEPT5	Influenza	SNAP25, PARK2	n/d	Up-regulated during H5N1 infection	[51]
	Influenza	n/d	n/d	Up-regulated during H5N1 infection	[53]
	Influenza	SEPT2, SEPT6, SEPT7, SEPT11, CRMP2, Tubulin, HSP	n/d	Expressed during H5N1 infection	[54]
SEPT6	MDV	n/d	n/d	Down-regulated during MDV infection – linked with oncogenesis	[50]
	HCV	hnRNP A1	NS5b	Involved as a scaffolding protein bound to NS5b and hnRNP A1, facilitating viral RNA replication	[58]
SEPT7	VACV	SEPT2, SEPT9 & SEPT11 (Complex)	n/d	Antiviral against VACV	[33]
	ZIKV	SEPT2	NS2B3	Down-regulated during ZIKV infection – disrupting cytokinesis	[80]
	HIV	n/d	Tat	Inducing cytoskeletal changes in Sertoli cells during HIV-1 infection	[85]
SEPT8	Nodavirus	n/d	n/d	Up-regulated during Nodavirus infection in brain of sea bream – regulating immune responses	[44]
SEPT9	VACV	SEPT2, SEPT7 & SEPT11 (Complex)	n/d	Antiviral against VACV	[33]
	MDV	n/d	n/d	Down-regulated during MDV infection	[50]
	HCV	PtdIns5P	n/d	Up-regulated during HCV infection	[64]
	HIV	n/d	n/d	Up-regulated in CD4+ T cells during infection	[83]
SEPT11	VACV	SEPT2, SEPT9 & SEPT7 (Complex)	n/d	Antiviral against VACV	[33]
	VACV	n/d	n/d	Antiviral against VACV	[35]

Listed are the known septins that are involved in the replication cycle of viruses, some of which exhibit antiviral or proviral effects, up-regulated or down-regulated protein expression, and interact with viral proteins that bring about pathogenesis. n/d corresponds to no data available from the study cited.

This GTP-binding proteins, a unique component of the cytoskeleton, hold valuable insights into understanding host-pathogen interactions during entry, exit, and movement within the hosts' cells. Through the integration of cell biology, pathophysiology, and biochemistry studies, our comprehension of this family of proteins will deepen, enhancing our ability to combat pathogens without incurring unnecessary time, costs, and effort. Targeting specific septin subtypes and functions, especially those contributing to viral replication, shows promise as an approach for therapeutic target development. In addition, investigations of their pathogenesis, mechanisms, and the pathways through which septins interact, particularly with viral components, may reveal a novel avenue for effectively treating viral infections.

## Abbreviations

AIDS: Acquired immunodeficiency syndrome
CEV: Cell-associated enveloped virus
CRMP2: Collapsin response mediator protein 2
DNA: Deoxyribonucleic acid
GaHV-2: Gallid herpesvirus 2
H5N1: Influenza A virus subtype H5N1
HCV: Hepatitis C virus
HHV-8: Human herpesvirus 8
HIV: Human immunodeficiency virus
hnRNPA1: heterogeneous nuclear ribonucleoprotein A1
HPAI: Highly pathogenic avian influenza
HSP: Heat shock protein
Hsp-70: Heat shock protein 70
KSHV: Kaposi’s sarcoma-associated herpesvirus
LD: Lipid droplets
MD: Marek’s disease
MDV: Marek’s disease virus
n/d: No data available
NF-κB: Nuclear factor κB
NS2B3: Non-structural protein 2B3
NS5b: Non-structural protein 5B
PARK2: Parkinson protein 2 E3 ubiquitin protein ligase isoform 3
pK15: Non-structural membrane protein K15
pre-mRNA: precursor messenger RNA
PtdIns5p: Phosphatidylinositol 5-phosphate
RNA: Ribonucleic acid
RNAi: RNA interference
SEPT1: Septin 1
SEPT2: Septin 2
SEPT3: Septin 3
SEPT4: Septin 4
SEPT5: Septin 5
SEPT6: Septin 6
SEPT7: Septin 7
SEPT8: Septin 8
SEPT9: Septin 9
SEPT10: Septin 10
SEPT11: Septin 11
SNAP25: Synaptosome-associated protein of 25 kDA
TNF-α: Tumor necrosis factor α
UNAIDS: Joint United Nations Programme on HIV/AIDS
VACV: Vaccinia virus
ZIKV: Zika virus
